# Opportunistic Cardiovascular Assessment During Lung Cancer Screening with Low-Dose Computed Tomography: A New Window for Prevention

**DOI:** 10.3390/arm94050075

**Published:** 2026-10-19

**Authors:** Katarzyna Sienkiewicz, Jadwiga Fijałkowska, Małgorzata Jelitto, Jakub Byczkowski, Michał Koliński, Edyta Szurowska, Witold Rzyman

**Affiliations:** 1 2nd Division of Radiology, Faculty of Health Sciences with the Institute of Maritime and Tropical Medicine, Medical University of Gdańsk, University Clinical Center, Mariana Smoluchowskiego 17, 80-214 Gdańsk, Poland; jfijalkowska@gumed.edu.pl (J.F.);; 2 Department of Thoracic Surgery, Faculty of Medicine, Medical University of Gdańsk, University Clinical Center, 80-214 Gdańsk, Poland

**Keywords:** lung cancer screening, low-dose computed tomography, incidental cardiovascular findings, coronary artery calcification, cardiovascular risk assessment, opportunistic screening, atherosclerosis, aortic calcification, Poland

## Abstract

**Highlights:**

**What are the main findings?**

- Low-dose CT (LDCT) performed for lung cancer screening incidentally visualises the heart and thoracic vessels, offering opportunistic cardiovascular risk information at no extra radiation or cost.
- Coronary artery calcification, aortic valve calcification, ascending aortic dilatation, moderate-to-large pericardial effusion and pulmonary artery enlargement are the incidental cardiovascular findings on screening LDCT with a defined downstream action, the prognostic evidence being strongest for coronary and aortic valve calcification; mitral annular and thoracic aortic calcification are risk-informative markers that should reinforce preventive care but do not by themselves trigger referral.

**What are the implications of the main findings?**

- A nationwide LDCT screening programme enters Poland’s guaranteed-benefits basket on 1 October 2026 (Dz.U. z 2026 r. poz. 976); pulmonologists, thoracic surgeons, general practitioners and cardiologists will increasingly need a shared, practical framework for interpreting these findings.
- Structured reporting, proportionate referral thresholds and coordinated pulmonology–cardiology–primary care pathways are needed to translate incidental imaging findings into measurable cardiovascular prevention; whether such reporting improves cardiovascular outcomes has not yet been demonstrated and should be tested in implementation studies.

**Abstract:**

Population-based lung cancer screening with low-dose computed tomography (LDCT) is expanding across Europe, including a publicly funded Polish programme starting in October 2026. Because screening-eligible individuals share substantial tobacco-related cardiovascular risk, LDCT also offers cardiovascular risk assessment without additional radiation. We conducted a narrative review of the PubMed literature published up to September 2026, prioritising guidelines, lung cancer screening cohorts and systematic reviews. We summarise the cardiovascular findings on non-contrast, non-electrocardiography (ECG)-gated LDCT: coronary artery, aortic valve, mitral annular and thoracic aortic calcification, ascending aortic dilatation, pericardial effusion, and pulmonary artery enlargement, as well as investigational cardiac volumetry, epicardial adipose tissue and artificial intelligence (AI)-assisted quantification. We grade each finding by reportability, defined downstream action and supporting evidence, and align reporting thresholds with the European Respiratory Society/European Society of Thoracic Imaging (ERS/ESTI) statement on incidental findings. LDCT cannot replace dedicated cardiac imaging or establish haemodynamic diagnoses. The prognostic value of coronary and aortic valve calcification and of ascending aortic dilatation is established, whereas the evidence for mitral annular calcification and pulmonary artery enlargement is more limited; observational data indicate that reporting these markers changes preventive management, but whether this improves cardiovascular outcomes has not yet been demonstrated. Standardised reporting, proportionate thresholds, and coordinated pathways linking radiology, pulmonology, primary care and cardiology are needed to translate incidental cardiovascular findings into scalable prevention and should be evaluated in implementation studies.

## 1. Introduction

Population-based lung cancer screening with low-dose computed tomography (LDCT) is expanding across Europe and globally, supported by robust evidence from the National Lung Screening Trial (NLST) and the NELSON trial showing a reduction in lung cancer mortality among high-risk individuals [1,2]. Europe’s Beating Cancer Plan and the subsequent EU Council Recommendation [3] on cancer screening have further encouraged Member States to evaluate and implement organised LDCT-based lung cancer screening programmes. Several European countries are therefore launching, piloting, or expanding national and regional screening initiatives. Poland is entering a decisive phase of this process. A regulation of the Minister of Health amending the regulation on guaranteed healthcare services, published on 22 July 2026, introduces a publicly funded LDCT-based lung cancer screening into the national basket of guaranteed health services, with contracts for its delivery permitted from 1 October 2026, following a positive recommendation of the President of the Agency for Health Technology Assessment and Tariff System (AOTMiT, Recommendation No. 98/2025) [4,5]. The programme will primarily target adults aged 55–74 years with a smoking history of at least 20 pack-years who currently smoke or quit within the preceding 15 years, with extended eligibility for individuals aged 50–54 years who have additional risk factors [4]. The screening-eligible population is characterised by a high burden of tobacco exposure, which constitutes the dominant shared driver of both lung cancer and cardiovascular disease: the individuals most likely to attend lung cancer screening are also among those at highest cardiovascular risk.

The timing is pertinent in view of Poland’s disease burden. The Ministry of Health’s regulatory impact assessment accompanying the above regulation cites over 20,700 new bronchus and lung cancer cases in 2022 and over 26,000 deaths attributed to these cancers [6]. In parallel, cardiovascular disease remains the leading cause of death in Poland, with diseases of the circulatory system accounting for nearly 37% of all deaths in 2023 [7]. Smoking also amplifies cardiometabolic risk, including a higher likelihood of type 2 diabetes, further increasing overall cardiovascular risk in screening participants [8,9].

A crucial and often underutilised consequence of this overlap is that LDCT performed for lung cancer screening provides a pragmatic platform for opportunistic cardiovascular assessment without additional radiation or a separate imaging pathway. Cardiovascular abnormalities are among the most clinically consequential incidental findings encountered in lung cancer screening cohorts. In the NLST, coronary artery calcifications (CAC) were among the most frequently detected significant incidental findings [10,11], and existing reporting frameworks already anticipate this interface. Guidance from the American College of Radiology (ACR) and the European multi-society (ERS/ESTI) statement supports a structured approach to incidental findings on low-dose CT. Commonly reported cardiovascular findings include coronary artery calcification, aortic valve calcification, ascending thoracic aortic dilatation, pericardial effusion, and pulmonary artery enlargement, with predefined thresholds used to guide referral and follow-up [12,13]. It should be noted that non-contrast, non-electrocardiography (ECG)-gated LDCT is not a substitute for dedicated cardiac imaging; cardiovascular information derived from it is mainly risk-informative rather than diagnostic.

With nationwide LDCT screening programmes entering implementation in multiple European countries, pulmonologists leading these programmes, alongside cardiologists and primary care physicians, will increasingly encounter structured cardiovascular reports and need to understand their clinical implications. Recent radiological reviews have summarised these findings in detail [14,15]; this review addresses their interpretation by pulmonologists and primary care physicians in the setting of a newly reimbursed national programme. Translating incidental cardiovascular information into measurable benefit requires a shared understanding of what can and cannot be inferred from LDCT, pragmatic referral and follow-up thresholds, and clear pathways linking radiology reports to primary care and cardiovascular prevention. If designed thoughtfully, oncology-led screening programmes can serve as a vehicle for earlier cardiovascular risk identification and prevention at scale.

## 2. Aim and Scope of This Review

This narrative review aims to equip pulmonologists and other clinicians involved in lung cancer screening programmes with a practical framework for opportunistic cardiovascular assessment on non-contrast, non-ECG-gated LDCT. We address the findings with the established evidence base: coronary artery calcification, aortic valve calcification, mitral annular calcification, thoracic aortic disease, pericardial effusion and pulmonary artery enlargement, alongside investigational metrics and the emerging role of artificial intelligence (AI)-assisted quantification. For each finding we specify whether it should be reported, whether a defined downstream action exists, and what type of evidence supports that action. We discuss implications of the expanding landscape of population-based LDCT screening for translating opportunistic imaging findings into scalable cardiovascular prevention.

## 3. Literature Search and Methodology

This narrative review was based on topic-specific PubMed searches performed by the authors responsible for the literature review, combining finding-specific terms (coronary, valvular and aortic calcification; aortic dilatation; pericardial effusion; pulmonary artery enlargement; cardiac volumetry and epicardial adipose tissue; artificial intelligence) with chest CT, low-dose CT and lung cancer screening terms, without a lower date limit, up to January 2026. Reference lists of guidelines and statements were screened, European and Polish regulatory and programme documents were retrieved from official repositories, and AI-assisted literature identification (Claude, Anthropic: Sonnet 4.5 and Fable 5.1; ChatGPT, OpenAI: GPT-5) was used for predefined topical queries, with every suggested reference verified against its source. During revision the search strategy was formalised and re-run in full on 29 September 2026 to verify coverage and update the reference list. Priority was given to guidelines and consensus statements, lung cancer screening cohorts, randomised trials, systematic reviews and large population-based datasets with cardiovascular outcomes; studies from other populations or imaging modalities were retained only as context for reference values, thresholds or measurement limitations. Case reports, conference abstracts and letters without original data were excluded. Consistent with the narrative design, sources were selected for relevance and representativeness, and the type of evidence supporting each reporting or referral action is indicated in Table 1.

## 4. Technical Scope and Cardiovascular Findings of Screening LDCT

Before addressing individual findings, it is important to define what screening LDCT can and cannot reliably show. Low-dose CT in lung cancer screening is performed without intravenous contrast and without ECG gating, using protocols optimised for pulmonary parenchyma rather than cardiac anatomy [16,17,18]. Cardiovascular information from screening LDCT is therefore opportunistic and risk-informative rather than diagnostic [12,13,19]. It can identify macroscopic calcifications (e.g., coronary and valvular calcium) and approximate large-vessel calibre (e.g., ascending aorta, main pulmonary artery) [12,13,19,20], but it cannot reliably grade coronary stenosis, characterise non-calcified plaque, or determine aortic valve stenosis severity [16]. Accordingly, findings should be reported within structured frameworks and used to prompt cardiovascular risk assessment and targeted follow-up when appropriate, while avoiding overinterpretation and unnecessary downstream testing [12,13,19]. We distinguish three tiers of findings (Table 1): (A) reportable findings for which a defined downstream action exists; (B) reportable, risk-informative findings for which no specific action beyond cardiovascular risk-factor optimisation is recommended; and (C) investigational metrics that are not currently recommended for routine reporting. For each finding, Table 1 also indicates the reporting trigger, the type of evidence supporting the proposed action and whether the recommendation is primarily evidence-based or expert-informed. The following sections address each major finding category in turn.

**Table 1:** Actionability grading of cardiovascular findings on lung cancer screening LDCT: reporting trigger, recommended action, type of supporting evidence and basis of the recommendation.

Finding	Tier	Reporting Trigger/Threshold	Recommended Action	Type of Supporting Evidence	Basis of Recommendation
Coronary artery calcification	A	Any visible CAC; visual grade (none/mild/moderate/severe)	Communicate to primary care; cardiovascular risk assessment and risk-factor optimisation; symptom-driven cardiology referral only; no routine further imaging	Prognosis: screening cohorts (NLST, NELSON, Polish cohort) [21,22,23,24]; incremental value over age and smoking [23]; management change: observational (statin prescribing) [25,26]; outcomes: none	Evidence-based (prognosis); multi-society statements [12,13,19]; expert-informed (pathway)
Aortic valve calcification	A	Moderate or severe visual grade	Symptom screening; transthoracic echocardiography; cardiology/valve pathway if significant AS confirmed	Association with echocardiographic AS in screening and non-gated CT cohorts [27,28,29,30]; management change: observational [28]; outcomes: none	Evidence-based (association); statements [13,19]; expert-informed (threshold)
Ascending aortic dilatation	A	Diameter ≥ 45 mm (ERS/ESTI); below 45 mm not reported; borderline values re-measured perpendicular to the vessel axis	Referral for further assessment according to local pathways; confirmation, surveillance and intervention thresholds per ESC aortic guidelines	Thresholds: consensus statement [13] and aortic guidelines derived from dissection-risk data in non-screening populations [31,32]; screening-specific: cost-effectiveness modelling only [33]; outcomes: none	Expert-informed/extrapolated
Pericardial effusion	A	Moderate or large effusion (qualitative)	Clinical assessment and echocardiography; urgent communication if large, new or with signs of haemodynamic compromise; consider malignancy	Consensus statement [13]; no screening-specific outcome data	Expert-informed
Pulmonary artery enlargement	A (conditional)	MPA ≥ 31 mm (ACR, as cited by ERS/ESTI) or PA:Ao > 0.9, particularly with ancillary CT signs	Isolated borderline value without symptoms or ancillary signs: risk-informative; with ancillary signs or disproportionate dyspnoea: clinical assessment, echocardiography, PH centre only within structured algorithm	Thresholds: consensus statements [12,13], general-population reference values and PH guideline [34,35]; prognosis: single screening cohort [36]; outcomes: none	Expert-informed (threshold and pathway); limited evidence (prognosis)
Mitral annular calcification	B	Any; visual grade (optional reporting)	Reinforce cardiovascular risk-factor optimisation; no specific referral	Prognosis: general-population cohorts only [37,38,39,40]; no screening cohort; no intervention data	Evidence-based (association, non-screening); expert-informed (no action)
Thoracic aortic calcification	B	Presence/extent (optional reporting)	Reinforce global cardiovascular prevention; no specific referral	Prognosis: screening cohorts [24,41] and general population [42]; no intervention data	Evidence-based (association); expert-informed (no action)
Cardiac chamber volumetry, LV mass, epicardial adipose tissue, AI-derived composite scores	C	Not recommended for routine reporting	Research use; prospective external validation required before clinical use	Prognosis: retrospective AI analyses of screening cohorts [43,44,45,46,47,48]; no validated LDCT-specific thresholds; no management or outcome data	Investigational

Tier A, reportable finding with a defined downstream action; Tier B, reportable, risk-informative finding for which no specific action beyond risk-factor optimisation is recommended; Tier C, investigational metric not recommended for routine reporting. AI, artificial intelligence; AS, aortic stenosis; CAC, coronary artery calcification; LDCT, low-dose computed tomography; LV, left ventricular; MPA, main pulmonary artery; NLST, National Lung Screening Trial; PA:Ao, pulmonary artery-to-aorta ratio; PH, pulmonary hypertension.

## 5. Coronary Artery Calcification on Screening LDCT

Coronary heart disease (CHD) may manifest catastrophically. In the Framingham cohort, 13–20% of coronary syndromes presented as sudden death depending on age, and approximately 60% of sudden CHD deaths occurred in individuals without previously recognized clinical heart disease [49]. Likewise, in Atherosclerosis Risk in Communities (ARIC) community surveillance, 32.6% of CHD deaths were sudden, and among those, 63.5% occurred in individuals without a prior CHD diagnosis, underscoring the potential public health value of opportunistic cardiovascular risk stratification in high-risk screening settings [50]. These observations provide the epidemiological rationale for opportunistic CAC detection in lung cancer screening populations, who share many of the same risk characteristics.

In lung cancer screening, coronary arteries are not the primary target of image optimisation. Because LDCT is non-contrast and non-ECG-gated, coronary segments may be partially blurred by cardiac motion, and image noise may be higher than in dedicated cardiac protocols [21,51]. These factors can reduce conspicuity of small or focal calcifications and, conversely, can make dense calcium appear more extensive because of partial-volume and blooming effects. As a result, agreement with dedicated ECG-gated calcium-scoring CT, while generally good, is not exact at the individual-patient level [51].

## 6. Visual CAC Assessment on LDCT: Methodology and Limitations

Current screening pathways favour visual, semi-quantitative CAC grading rather than formal Agatston quantification [12,13,19]. A widely adopted approach is a visual grade of none, mild, moderate, and severe, recommended for routine reporting on non-gated thoracic CT to communicate cardiovascular risk in a way that is feasible at scale [12,13,19]. An alternative method is ordinal vessel-based scoring, in which calcification is graded from 0 to 3 in each major coronary artery: the left main coronary artery, left anterior descending artery, right coronary artery, and left circumflex artery. The scores are summed to give a total score from 0 to 12, categorized as absent (0), mild (1–3), moderate (4–6), or severe (7–12) [52] (Figure 1).

From a clinical perspective, visual scoring systems may yield inconsistent results in individual patients. They are observer-dependent [51], and performance is weakest at the low end of disease burden, where partial-volume effects are most pronounced [53]. They can also be misleading in specific anatomic situations (e.g., hypoplastic vessels, anatomical variants), where some vessels may be systematically over- or under-represented in an ordinal framework. Qualitative categories do not directly translate into established treatment thresholds unless the clinical pathway defines how each grade should modify prevention decisions and downstream management [54,55].

## 7. Agatston Scoring on Non-Gated LDCT: Feasibility and Caveats

Agatston scoring can be performed on non-gated LDCT in research settings and in some advanced clinical workflows, and correlations with ECG-gated calcium scoring are typically high [17,54]. However, variability between techniques can be substantial [56]. Agatston values derived from non-gated chest CT should not be treated as interchangeable with standardized ECG-gated scoring [57]. Systematic differences may also arise from acquisition and reconstruction parameters, including slice thickness and volume averaging [58]. Meta-analytic comparisons show excellent overall agreement with ECG-gated CT but also demonstrate misclassification and underestimation of higher CAC categories in a subset of scans, with agreement influenced by reconstruction parameters such as slice thickness and kernel [59].

## 8. Clinical Interpretation of CAC on Screening LDCT

LDCT-derived CAC (visual or quantitative) is a marker of atherosclerotic disease presence and burden, and its detection on screening LDCT carries independent prognostic value for long-term mortality [22]. In NLST participants, CAC assessed on LDCT using both visual and Agatston-based methods was strongly associated with coronary heart disease death and all-cause mortality, with simple visual approaches performing comparably to quantitative methods for risk stratification [21]. The finding is consistent across screening cohorts, including NELSON [23] and a Polish screening cohort [22]. In NELSON, modified Agatston categories improved risk classification over a model based on age, pack-years and smoking status, i.e., the variables that define screening eligibility [23]. In a separate screening cohort, a prediction model combining coronary and aortic calcium volume from screening LDCT was derived and validated for cardiovascular events [24]. At the same time, LDCT provides no information on non-calcified plaque and cannot rule out obstructive coronary artery disease [60]. Absence of visible CAC on a non-gated, low-dose scan should therefore not be used to exclude coronary artery disease in symptomatic patients [61]. The appropriate clinical framing is: LDCT identifies coronary artery disease risk when CAC is present, but does not characterize stenosis severity or plaque composition, and symptom-driven diagnostic pathways remain unchanged.

An additional consideration in screened populations is the so-called statin paradox: statin therapy may increase measured coronary calcification while reducing cardiovascular risk and events. Consequently, CAC severity on LDCT should be interpreted in clinical context (including lipid-lowering therapy) and should not be used in isolation to infer disease progression or to escalate care inappropriately in treated patients [62].

A further practical pitfall is the presence of coronary stents. On non-contrast datasets, metallic stents and dense calcification can be difficult to distinguish without clinical history, potentially leading to overestimation of CAC severity. Noteworthy, CAC scoring is generally not indicated in patients with prior revascularization, as stent material renders the assessment unreliable and clinically uninformative [63] (Figure 1).

Opportunistic CAC assessment performed on screening LDCT provides clinically meaningful prognostic information at a population level, but its interpretation requires awareness of technical limitations, concomitant therapies, and prior interventional history.

## 9. Aortic Valve Calcification on Screening LDCT

Degenerative calcific aortic stenosis (AS) is common in older adults and increases steeply with age. In the population-based Tromsø Study, echocardiographic AS prevalence rose from 0.2% in the sixth decade to 1.3% in the seventh and 3.9% in the eighth decade, reaching approximately 10% at 80–89 years [64]. A meta-analysis restricted to individuals older than 75 years reported a pooled prevalence of 12.4% for any AS and 3.4% for severe AS [65]. Within the 55–74-year age range targeted by the Polish programme, the expected population prevalence of AS is therefore in the order of 1–4%, rising across the eligible age span. In the previously published MOLTEST BIS screening cohort from Gdańsk (6631 participants aged 50–80 years) [30], any aortic valve calcification was present in 13.1%; participants with a modified aortic valve calcium score ≥ 900—a prespecified referral threshold based on the 2017 ESC/EACTS guidelines—were referred for echocardiography, and AS was confirmed in 49 individuals, i.e., 0.7% of the whole cohort (mild 0.2%, moderate 0.3%, severe 0.2%), 55% of whom—including 6 of the 13 with severe AS—had been unaware of their valve disease [30]. The lower figure represents the yield of a calcium-triggered echocardiography pathway rather than prevalence: mild AS with a calcium score below the referral threshold was not captured. A subsequent analysis of the same cohort identified calcium score cut-offs of 1758 for at least moderate and 2665 for severe AS [66]. Although AS may remain asymptomatic for years, prognosis becomes poor once symptoms develop in severe AS without valve intervention [67]. Sudden death can occur even without preceding symptoms, with contemporary registry data indicating risk in conservatively managed severe AS of up to 7.2% [68].

On non-contrast, non-ECG-gated LDCT performed for lung cancer screening, aortic valve calcification (AVC) is usually conspicuous and can be reported using a visual grade (e.g., none, mild, moderate, or severe) [13,19] (Figure 2).

This approach is inherently semi-quantitative and observer-dependent, with better reproducibility at higher calcification burdens [27]. In lung screening cohorts, visual AVC grading on LDCT shows clinically useful association with echocardiographic AS severity and may help identify individuals with previously unrecognized, asymptomatic or minimally symptomatic clinically significant AS who warrant targeted transthoracic echocardiography [28]. Visual ordinal AVC grading on routine non-gated chest CT has also been shown to alter clinical management [28]. Although echocardiography remains the gold standard for assessment of AS, quantitative CT aortic valve calcium scoring (CT-AVC; Agatston units) can be derived from non-contrast CT and may be useful in patients with discordant echocardiographic findings, with validated diagnostic thresholds established for dedicated ECG-gated protocols [69]. CT-AVC values obtained from screening LDCT should not be treated as interchangeable with standard gated measurements, as motion artifacts, reconstruction parameters, and partial-volume effects may bias absolute values [29]. On non-gated LDCT, fine anatomic separation of leaflet from peri-annular calcification may also be less reliable, impairing diagnostic specificity [70,71]. Recent European Society of Cardiology/European Association for Cardio-Thoracic Surgery (ESC/EACTS) recommendations have adopted lower sex-specific calcium-burden thresholds for severe AS, reinforcing the need for heightened vigilance when AVC is present [72]. In practical terms, incidental moderate-to-severe AVC on screening LDCT should prompt symptom screening and referral for transthoracic echocardiography [13,30]. AVC on screening LDCT should therefore be regarded not as an incidental finding to be dismissed, but as a potential entry point for timely echocardiographic evaluation in a population where significant aortic stenosis may otherwise go undetected.

## 10. Mitral Annular Calcification

Mitral annular calcification (MAC) is typically conspicuous on non-contrast, non-ECG-gated LDCT and, in screening practice, is best reported qualitatively (e.g., none, mild, moderate, and severe) [19] (Figure 2). Although LDCT cannot assess hemodynamic severity (mitral stenosis and/or regurgitation), MAC is recognized as a marker of degenerative and atherosclerotic disease burden: population studies link MAC to higher cardiovascular morbidity and mortality and to cerebrovascular events [37,38], and MAC is also associated with incident atrial fibrillation [39,40]. These associations derive from general-population cohorts; no lung cancer screening cohort has yet linked MAC on LDCT to outcomes, and no intervention study has targeted MAC. Current European guidance on incidental findings in lung cancer screening LDCT (ERS/ESTI, 2023) [13] does not recommend any specific clinical follow-up action based on MAC alone, reflecting the limited evidence for MAC-directed intervention in asymptomatic screening populations. MAC identified on screening LDCT should therefore be regarded as a marker of overall cardiovascular risk burden rather than an independent trigger for downstream investigation or referral. In clinical practice, its presence may reinforce the importance of cardiovascular risk factor optimization, though no specific management action is currently mandated by screening guidelines.

## 11. Thoracic Aortic Calcification

Standard non-contrast, non-ECG-gated LDCT for lung cancer screening routinely covers the whole thoracic aorta (ascending aorta, arch, and descending aorta) and often the proximal origins of supra-aortic vessels, depending on scan range [73]. The risk associated with thoracic aortic calcification (TAC) has been assessed in the context of both screening LDCT and coronary CT angiography (CCTA) [74]. There is no clear consensus regarding the methodology for assessing TAC; approaches to quantification vary, including binary assessment and modified Agatston-based methods [75] (Figure 2). In one lung cancer screening study, TAC scores provided additional prognostic information beyond CAC and established risk factors, particularly for predicting non-CHD cardiovascular mortality and all-cause mortality [41], and aortic calcium volume contributed to a validated screening-CT-based model predicting cardiovascular events [24]. In a large cohort study, TAC of 500 (Agatston score) or more, compared with TAC of 0, was significantly associated with a higher risk of cardiovascular disease and all-cause mortality, with the strongest association observed among individuals with a negative CAC score [42]. These observations support using TAC on screening LDCT primarily for cardiovascular risk stratification and targeted prevention, rather than as a stand-alone diagnostic test.

## 12. Ascending Aortic Dilatation

Ascending aortic dilatation may be detected incidentally and can remain asymptomatic for years; age and smoking exposure represent relevant risk contexts, and longitudinal data in screened smokers suggest that average thoracic aortic growth is small but measurable over time [76]. Key limitations of LDCT for proximal aortic assessment include motion artifacts in the aortic root and ascending aorta due to the absence of ECG gating, and non-contrast diameter measurement, where the lumen-wall interface is less distinct and diameters may be overestimated if wall thickness is inadvertently included, or if the measurement is not perpendicular to the vessel axis. For triage in screening programs, the ERS/ESTI statement recommends that a thoracic aortic diameter below 45 mm is not reported, that a diameter ≥ 45 mm is reported, and that participants with an aorta ≥ 45 mm are referred for further assessment according to local guidelines and pathways [13]; dedicated follow-up imaging appears to be most cost-effective from ≥50 mm [33] (Figure 2). In this review, and in Table 1, we adopt the ERS/ESTI threshold. First, because axial non-contrast measurement can overestimate the diameter, borderline values should be re-measured perpendicular to the vessel axis before an aorta is reported as ≥45 mm. Second, once a participant is referred, diagnostic confirmation (ECG-gated CT or MR angiography, or echocardiography of the aortic root), the surveillance interval and any threshold for intervention are determined by cardiology according to the ESC aortic guidelines [32], not by the screening programme. The 45 mm threshold is consensus-based; the only screening-specific evidence is cost-effectiveness modelling [33], and no outcome data exist for aortic referral triggered by screening LDCT. Bicuspid aortic valve, a common cause of ascending dilatation in this age group, cannot be reliably assessed on non-gated, non-contrast LDCT, although the coexistence of ascending aortic dilatation with aortic valve calcification, particularly at the younger end of the screening age range, should raise this possibility; its diagnosis, and the lower thresholds that then apply, rest on dedicated cardiac imaging according to the ESC guidelines [32].

## 13. Pericardial Effusion on Screening LDCT

Pericardial effusion may be seen on non-contrast LDCT as a fluid collection within the pericardial sac. In the lung cancer screening setting, reporting should remain pragmatic. The 2023 ERS/ESTI statement recommends that trivial or small amounts of pericardial fluid should not be reported, whereas moderate or large effusions should be reported and referred for clinical assessment and echocardiography [13]. On non-ECG-gated LDCT, effusion size can only be assessed approximately; therefore, echocardiographic thresholds should not be directly applied to CT. Reports should describe moderate or large effusions qualitatively. Substantial effusions, particularly when new or associated with cardiac decompensation, should be communicated promptly. In screening participants, malignant pericardial involvement should remain part of the differential diagnosis, although other causes are also frequent. Recent reviews of non-contrast chest CT likewise list moderate or large pericardial effusion among findings warranting communication [14]; screening-specific data are essentially absent, and the recommendation rests on consensus alone.

## 14. Pulmonary Artery Enlargement on Screening LDCT

On non-contrast, non-ECG-gated LDCT, the main pulmonary artery (MPA) is usually well visualized and can be measured on axial images at the level of its bifurcation perpendicular to its long axis [34]. Reference values are sex-specific and vary with body size (Framingham 90th percentiles: 29 mm in men, 27 mm in women; PA-to-aorta (PA:Ao) ratio 0.9) [34], and the 2022 ESC/ERS pulmonary hypertension (PH) guideline lists an MPA diameter ≥ 30 mm and a PA:Ao ratio > 0.9 among CT signs suggestive of PH [35]. The ERS/ESTI statement itself does not define a cut-off but cites the ACR quick reference guide for lung cancer screening, which advises primary care evaluation for an MPA diameter of at least 31 mm [12,13]; in this review, and in Table 1, an MPA diameter ≥ 31 mm or a PA:Ao ratio > 0.9 is used as the reporting trigger because it is the value that radiologists in European programmes following the ERS/ESTI statement will apply in practice (Figure 3). The referral burden under this trigger has not been reported directly in a screening cohort, but it can be bounded: among 1949 smokers undergoing baseline screening LDCT, 4.2% had an MPA ≥ 34 mm (two standard deviations above the mean) and 6.9% a PA:Ao ratio ≥ 1.0 [77], whereas in a screening cohort of 777 participants assessed with the sex-specific Framingham cut-offs, 23% had PA enlargement, which independently predicted mortality after adjustment for age, smoking status, hypertension and emphysema severity (hazard ratio 1.83 for MPA diameter and 3.78 for the PA:Ao ratio) [36]. The proportion flagged by an MPA ≥ 31 mm or a PA:Ao ratio > 0.9 therefore lies between these values, which is why the trigger is conditional on ancillary signs or symptoms (Table 1). LDCT may also support aetiological assessment by revealing parenchymal disease consistent with WHO group 3 PH (lung disease or hypoxia), and may occasionally show indirect signs of chronic thromboembolic pulmonary hypertension (CTEPH, WHO group 4), such as mosaic attenuation or peripheral wedge-shaped opacities, though dedicated contrast-enhanced imaging is required for definitive work-up [35,78]. Ancillary signs of right-sided pressure overload, including right-sided chamber enlargement and inferior vena cava dilatation, may strengthen clinical suspicion [35,78] (Figure 3).

It must be emphasized that an isolated MPA measurement does not establish PH, and PH cannot be diagnosed on LDCT; definitive diagnosis requires hemodynamic confirmation with right heart catheterization as part of a structured diagnostic algorithm [35]. MPA diameter is a non-specific parameter: it increases with age and body size, with hypoxic lung disease (emphysema and chronic obstructive pulmonary disease, interstitial lung disease), with obesity and with left heart disease, so that in a screening population selected for heavy smoking exposure a substantial proportion of enlargement reflects group 3 physiology or incidental variation rather than pulmonary arterial disease [35]. Measurement variability on non-gated LDCT (level and angle of measurement, respiratory phase, cardiac motion) can also move a borderline value across the threshold. Accordingly, an isolated borderline measurement without ancillary CT signs and without symptoms should be regarded as a risk-informative finding rather than as an indication for further testing, whereas MPA enlargement accompanied by ancillary signs, by dyspnoea disproportionate to spirometry, or by suspected CTEPH should prompt clinical assessment followed by echocardiography, with onward referral to a specialist centre and right heart catheterization only within the structured PH algorithm [35]. In the context of a lung cancer screening programme, pulmonologists should consider pulmonary vascular disease within the broader differential of dyspnoea or functional impairment in screening participants.

## 15. Beyond Routine Reporting: Investigational Cardiovascular Metrics on Screening LDCT

### 15.1. Cardiac Chamber Volumetry and LV Myocardial Mass

On non-contrast, non-ECG-gated LDCT, delineation of the endocardial border is constrained by the similar attenuation of blood and myocardium, image noise, and cardiac motion. Accordingly, chamber volumes and left ventricular (LV) myocardial mass, when reported, generally rely on automated AI-based segmentation rather than manual or visual assessment [43,79]. Despite the limitations related to the absence of ECG gating, LDCT-derived measures such as LV and left atrial volumetry and LV mass have shown prognostic value, particularly within multi-parameter predictive models, having been associated with higher all-cause and cardiovascular mortality and an increased risk of heart failure hospitalization [44]. At present, the absence of universally accepted cutoffs and clearly defined downstream pathways in lung cancer screening limits these measures largely to research use.

### 15.2. Epicardial Adipose Tissue (EAT) Quantification

EAT quantification on non-contrast chest CT, most commonly volume and, in some studies, attenuation as a surrogate of fat quality, is an actively investigated opportunistic biomarker [45]. Mechanistically, EAT is thought to exert adverse cardiovascular effects through paracrine and vasocrine secretion of pro-inflammatory and profibrotic mediators, and has been implicated in the pathogenesis of coronary artery disease, atrial fibrillation, and heart failure [80,81]. Across observational studies, automated EAT volume and attenuation have shown prognostic value beyond traditional risk factors and CAC, including in individuals without detectable CAC, and longitudinal EAT changes on serial LDCT have been associated with cardiovascular outcomes with reported sex-specific differences [45,46,47]. However, translation to routine clinical decision-making remains constrained by the non-ECG-gated nature of screening LDCT: motion artifacts, partial-volume effects at fat-soft tissue interfaces, and variability introduced by acquisition and reconstruction parameters (including tube voltage) can bias EAT estimates, and no validated thresholds or management pathways currently exist for EAT derived from non-gated protocols [82].

### 15.3. Artificial Intelligence and Automated Quantification in LDCT Screening

Lung cancer screening LDCT is particularly well suited for automation because it is performed at scale, with consistent anatomical coverage and a narrow range of dose-optimised acquisition protocols defined by programme technical standards, generating high-volume datasets for algorithm development and external validation [16,73]. This standardisation applies to coverage and dose, however, and not to reconstruction: slice thickness, kernel, tube voltage and iterative reconstruction differ between vendors and sites and, as discussed in the preceding sections, bias absolute CAC, AVC and EAT values relative to dedicated ECG-gated protocols. Quantitative thresholds validated for ECG-gated calcium scoring therefore cannot be transferred to LDCT, and AI outputs from LDCT should be interpreted against LDCT-specific reference data rather than gated cut-offs [57,59]. Deep-learning pipelines have already demonstrated accurate, rapid automated quantification of calcifications on LDCT, most notably coronary artery calcium (including Agatston-like outputs) and, in some approaches, multi-site calcification assessments encompassing the coronary arteries, thoracic aorta, and cardiac valves, potentially converting current largely visual reporting into reproducible quantitative metrics [83,84,85]. Beyond calcium, multi-parameter models derived from non-contrast chest CT (including CAC together with chamber volumetry and LV mass index) have shown incremental prognostic value and improved risk stratification compared with routine qualitative reporting [43], and radiomic descriptors of cardiac and aortic structure predicted cardiovascular mortality in NLST beyond the PREVENT risk equations and CAC [48]. External validation across scanners, protocols and populations remains limited: automated CAC categorisation showed almost perfect agreement between standard low-dose and ultra-low-dose tin-filter protocols acquired in the same breath-hold (weighted kappa 0.86) [86], but comparable data across vendors and reconstruction settings are scarce. For safe clinical deployment, automated pipelines should incorporate quality-control layers that flag motion artifacts, stents, low-confidence segmentations and out-of-range values for targeted human verification, and deliver results as structured outputs that can be integrated into radiology reports and interoperable data standards [87]. Evidence that automated measurements lead to appropriate clinical decisions is still lacking: the validation studies to date are retrospective and measure agreement or prognostic association, not whether AI-reported findings change management or outcomes. The same limitation applies more widely to machine-learning models in lung cancer: recently published classifiers built on small, single-source datasets report internal validation only, and their authors themselves call for testing on larger external cohorts [88]. Clinical decision thresholds and downstream pathways are therefore not yet established for AI-derived metrics obtained from LDCT, and outputs will still require clinical contextualization and confirmation [89].

## 16. Discussion

Lung cancer screening LDCT programmes create a scalable and repeated opportunity to improve cardiovascular prevention by leveraging opportunistic risk markers visible on non-contrast, non-ECG-gated scans, without additional radiation or a separate imaging pathway. As nationwide programmes enter implementation across Europe, the clinical value of cardiovascular information embedded in LDCT reports will depend critically on systematic reporting, acknowledgement, and clinical follow-up.

Pulmonologists, as the primary clinical leads of lung cancer screening programmes, are uniquely positioned at the interface between oncological and cardiovascular care in this population. Understanding the meaning and limitations of cardiovascular incidental findings, and knowing when to escalate versus when to simply intensify preventive care, is becoming an essential competency for screening programme coordinators. This highlights the need for predefined collaboration and referral pathways between lung cancer screening programmes and cardiology services for clinically relevant cardiovascular incidental findings.

The evidence supporting opportunistic cardiovascular assessment on LDCT is strongest at the level of prognosis. Across lung cancer screening cohorts—NLST [21], NELSON [23], a Dutch screening-based prediction cohort [24] and a Polish screening cohort [22]—CAC on non-gated LDCT independently predicted cardiovascular events and mortality, and in NELSON modified Agatston categories improved reclassification over age, pack-years and smoking status, i.e., over the variables that define screening eligibility [23]. More recently, AI-derived measures of cardiac and aortic structure from NLST scans predicted cardiovascular mortality beyond the PREVENT risk equations and CAC [48], and pulmonary artery enlargement independently predicted mortality after adjustment for smoking and emphysema severity [36]. European prevention guidelines already allow CAC scoring to be used as a risk modifier when treatment decisions are uncertain [55]; a formal demonstration that LDCT-derived CAC reclassifies Systematic COronary Risk Evaluation 2 (SCORE2 and SCORE2-OP) categories in screening participants is, however, still lacking.

The evidence that reporting these findings changes management is more limited but consistent in direction. In a multicentre US screening cohort, CAC on the LDCT report was associated with subsequent statin prescription (adjusted odds ratio 2.6), yet 60% of statin-eligible participants remained untreated at baseline [25]. In a further screening cohort, lipid-lowering therapy was prescribed in 60% of participants whose CAC was reported versus 45% when it was not [26]. Visual ordinal AVC grading on routine chest CT has likewise been shown to alter management [28]. In a UK screening programme with structured triage of incidental findings, most findings required no ongoing follow-up, while a minority led to diagnosis and intervention [90]. By contrast, the YESS randomised trial found that showing screening participants their own annotated CT images, including coronary calcification, in addition to co-located cessation support did not increase validated quit rates over standard support [91]—a reminder that a “teachable moment” does not automatically translate into behaviour change.

Whether opportunistic cardiovascular reporting within lung cancer screening improves cardiovascular outcomes has not been tested. The downstream consequences are not negligible: incidental findings generate cascades of further testing of uncertain net benefit [92], echocardiography referrals produce their own incidental findings, and preventive therapy may be started in people who would otherwise not have been treated. The American Thoracic Society research statement on incidental findings in lung cancer screening identifies these gaps—the effect of reporting on outcomes, and downstream costs and harms—as research priorities [93]. The window for prevention described in this review is therefore an established prognostic opportunity whose clinical benefit remains to be demonstrated, and programmes implementing structured cardiovascular reporting should build in prospective evaluation of referrals, downstream testing, therapy uptake and outcomes.

In parallel with the broader European roll-out, Poland is already generating local evidence for integrated, multi-disease prevention based on screening LDCT. The MULTIPREVENT cohort study in Gdansk, funded by the Medical Research Agency (ABM, project no. 2023/ABM/03/00036), is evaluating multimorbidity in high-risk smokers by combining multiparametric analysis of non-contrast chest LDCT (including pulmonary and cardiac findings, body composition, and upper-abdominal findings) with spirometry, blood pressure and anthropometric measurements, selected genetic and laboratory testing (including Lp(a) and HbA1c), and structured clinical and epidemiological data collection. Recruitment is ongoing, and the aim is to develop predictive models tailored to the Polish population and enable coordinated preventive interventions [94,95]. Such initiatives illustrate the broader potential for lung cancer screening infrastructure to serve as a platform for integrated disease prevention.

At a minimum, structured reporting should deliver a concise, clinically interpretable core set of findings paired with proportionate, pathway-based downstream suggestions, as proposed for Lung-RADS-based reporting of significant incidental findings [15]. The downstream impact, however, depends not only on reporting consistency but also on whether report recipients understand the meaning and limitations of these markers. Cross-specialty education, particularly in pulmonology and primary care, is essential to avoid both missed prevention opportunities and unnecessary diagnostic cascades. Standardised minimum datasets, agreed upon across radiology, pulmonology, primary care and cardiology, provide the foundation for this communication.

In many asymptomatic individuals with isolated calcification markers, the most effective and scalable response will be intensification of preventive care: lipid lowering, blood pressure control, diabetes management, and smoking cessation, without automatic escalation to advanced imaging. Specialist pathways should be reserved for patients who are symptomatic, have high-risk features, or show rapid progression (Table 1, Figure 4). In the pathways described in current screening statements [12,13], the LDCT report is not an isolated trigger: results are communicated by a clinician who takes a history, and it is the combination of a high-risk imaging marker with symptoms, uncontrolled risk factors or a defined high-risk feature—not the marker alone—that drives escalation.

This principle of proportionate response is central to sustainable programme design and avoids the risk of generating uncoordinated downstream testing and service overload.

AI-assisted quantification is likely to increase both the volume and granularity of cardiovascular information extracted from screening LDCT, converting predominantly visual assessments into reproducible quantitative metrics. However, a major implementation gap remains: decision thresholds and downstream pathways are not yet uniformly established for AI-derived metrics obtained from non-gated LDCT, and the validation performed so far is largely retrospective. AI-derived quantitative metrics will require prospective, external validation across scanners, vendors and LDCT protocols, with predefined decision thresholds and audit of the downstream clinical actions they generate, before routine implementation. Health systems planning LDCT roll-out should anticipate and prepare for this influx of quantitative findings, establishing governance frameworks that ensure outputs are clinically contextualised, quality-assured, and connected to evidence-based care pathways before wide-scale deployment.

## 17. Limitations

Several limitations of this review should be acknowledged. The narrative design allowed integration of clinically relevant evidence and current perspectives across a broad field, but did not include formal systematic review methods or meta-analysis, and some relevant studies may have been missed. The scope is focused on findings with the most established evidence base and clinical relevance in the context of population-based LDCT screening; it does not address all cardiovascular findings potentially visible on LDCT. The evidence base for some findings (e.g., MAC, thoracic aortic calcification) remains less robust than for CAC, and recommendations must therefore be interpreted as expert-informed guidance rather than evidence-based mandates. The reporting thresholds for aortic dilatation and pulmonary artery enlargement in particular are consensus-based, as made explicit in Table 1. No study has yet shown that reporting these findings within a lung cancer screening programme improves cardiovascular outcomes. Finally, the regulatory and reimbursement landscape for AI-assisted cardiovascular reporting on LDCT is evolving rapidly, and specific recommendations in this area may require updating as the field matures.

## 18. Conclusions

Population-based LDCT lung cancer screening creates a scalable opportunity to improve cardiovascular prevention by leveraging opportunistic risk markers, including coronary and valvular calcification, aortic dilatation, pericardial effusion, and pulmonary artery enlargement, visible on non-contrast, non-ECG-gated scans. The prognostic value of coronary and aortic valve calcification and of ascending aortic dilatation in screening cohorts is established, whereas the evidence for mitral annular calcification and pulmonary artery enlargement remains more limited; observational data indicate that reporting these markers changes preventive management, but that this improves cardiovascular outcomes remains a hypothesis to be tested, so the window for prevention described here is an opportunity rather than a demonstrated benefit. Real-world benefit will require standardised minimum core datasets in structured reports, proportionate follow-up recommendations, and education across pulmonology, radiology, cardiology, and primary care so that imaging markers translate into appropriate preventive action rather than unstructured testing. Pulmonologists leading these programmes have a critical role in ensuring that cardiovascular findings are embedded within coordinated, multidisciplinary follow-up pathways. AI is likely to amplify the availability of quantitative metrics, but its clinical utility will depend on establishing validated thresholds and integrated pathways that reliably connect findings with evidence-based care. Future implementation research should determine how such approaches can be adapted across different healthcare systems and whether they improve preventive therapy uptake, risk-factor control, and cardiovascular outcomes.

## Figures and Tables

**Figure 1:**
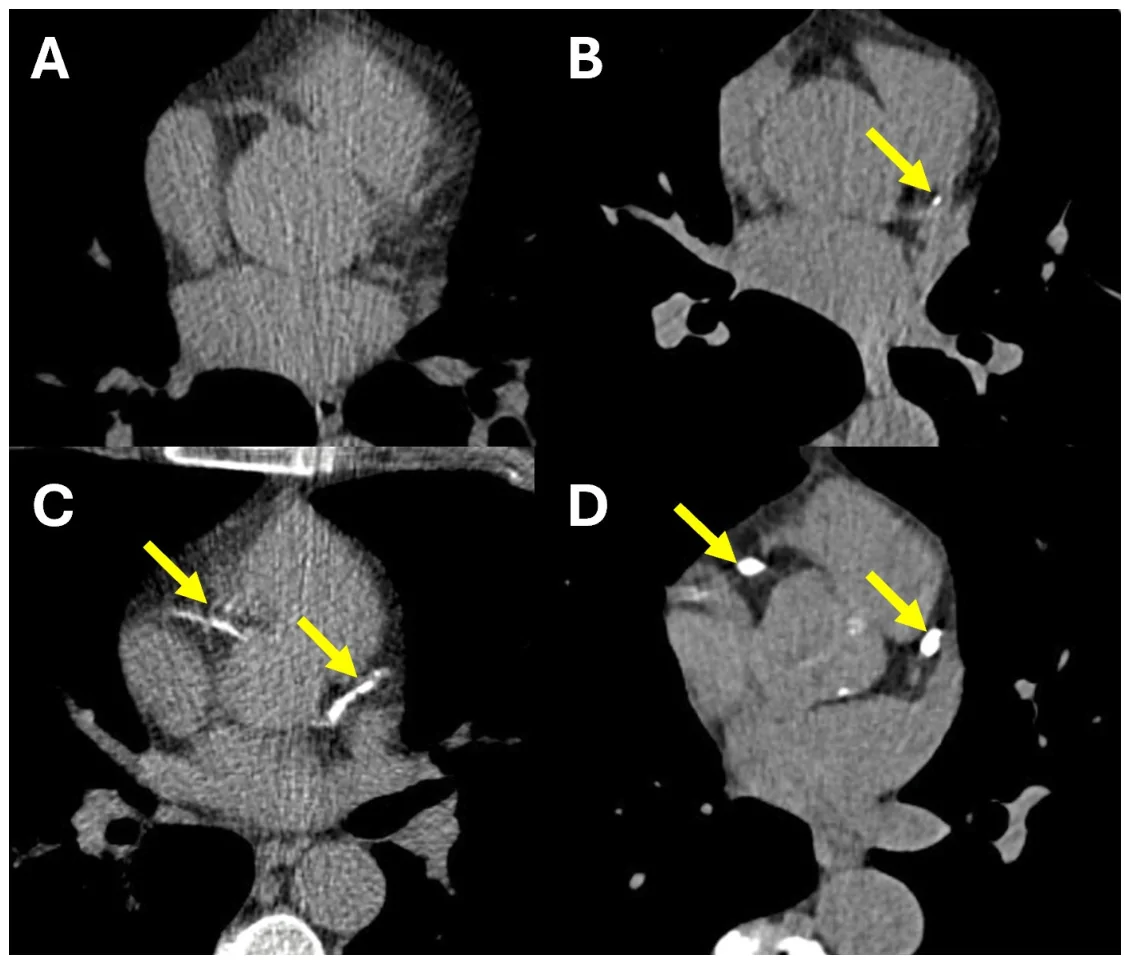
Visual grading of coronary artery calcification (CAC) on lung cancer screening low-dose computed tomography (LDCT). Axial non-contrast, non-ECG-gated images. (**A**) No CAC. (**B**) Mild CAC: focal calcification in the left anterior descending artery (arrow). (**C**) Severe CAC: extensive calcifications in the left anterior descending and right coronary arteries (arrows). (**D**) Coronary stents in the right coronary artery and left anterior descending artery (arrows).

**Figure 2:**
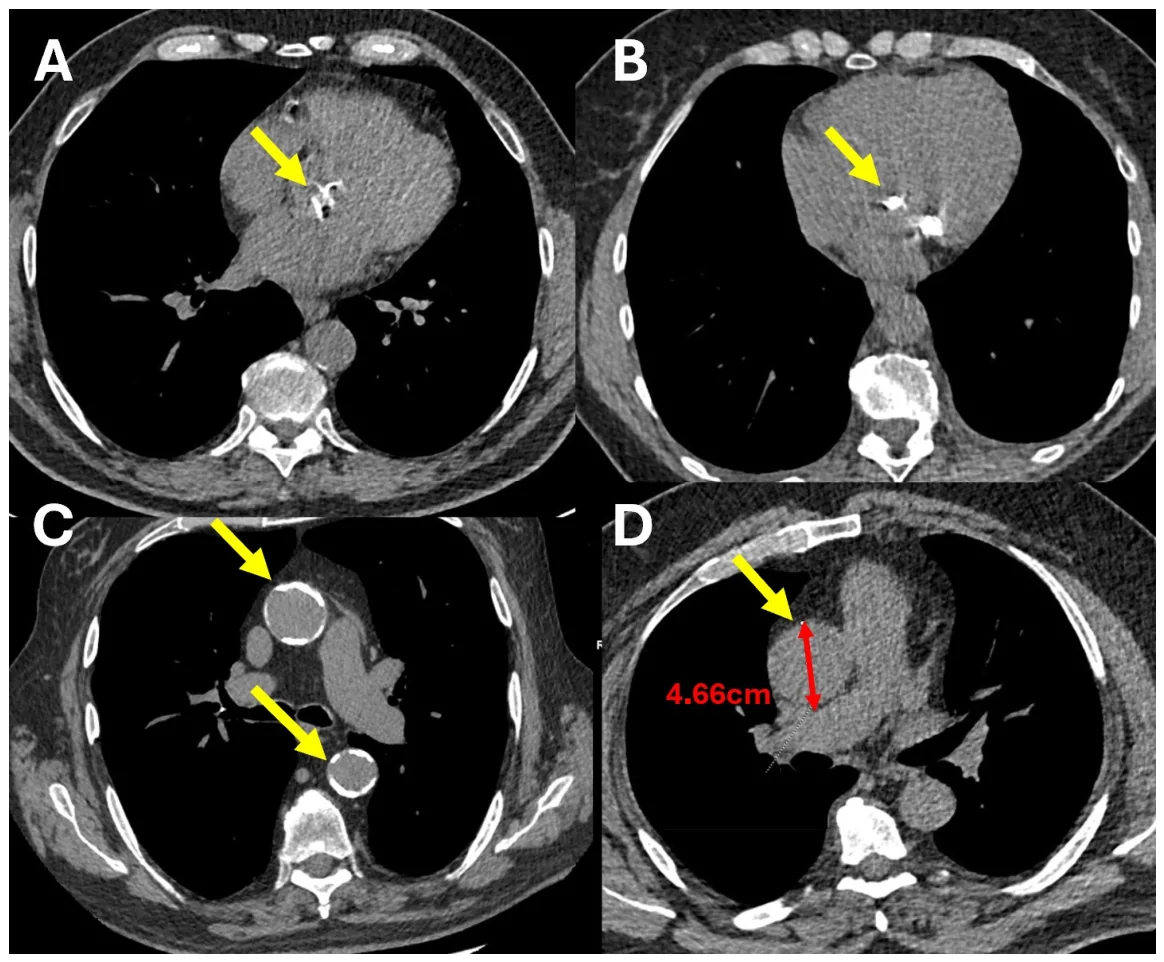
Opportunistic cardiovascular findings on lung cancer screening low-dose computed tomography (LDCT). Axial non-contrast, non-ECG-gated images. (**A**) Aortic valve calcification (AVC): dense calcification at the level of the aortic valve (arrow). (**B**) Mitral annular calcification (MAC): high-attenuation calcification at the mitral annulus (arrow). (**C**) Thoracic aortic calcification (TAC): calcification visible in the ascending and descending thoracic aorta (arrows). (**D**) Ascending aortic dilatation: markedly enlarged ascending aorta measuring 4.66 cm on axial non-contrast image (arrow).

**Figure 3:**
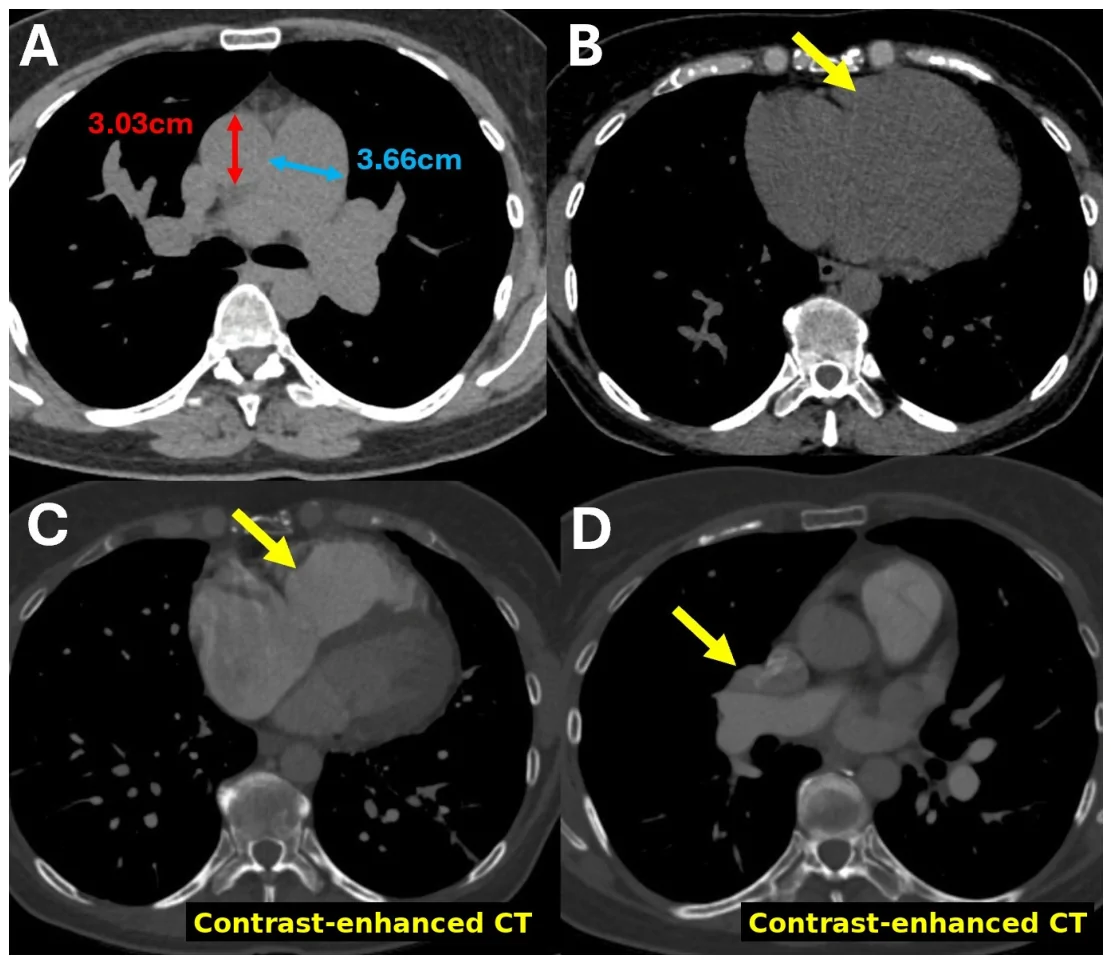
Pulmonary artery enlargement and ancillary signs of pulmonary hypertension (PH) on computed tomography (CT). (**A**) Non-contrast screening LDCT: enlarged main pulmonary artery (MPA) measuring 3.66 cm (arrow); PA-to-aorta ratio 1.21. (**B**) Non-contrast screening LDCT: right ventricular predominance (arrow). (**C**) Contrast-enhanced CT pulmonary angiography (clinical examination following the screening scan): confirmed right ventricular dilatation in the same patient (arrow). (**D**) Contrast-enhanced CT (clinical examination following the screening scan): anomalous drainage of the right upper pulmonary vein into the superior vena cava (arrow), a congenital left-to-right shunt. Panels (**C**,**D**) are contrast-enhanced clinical examinations, shown to illustrate what the screening scan alone (**A**,**B**) cannot depict: intracardiac anatomy, shunts and pulmonary emboli require contrast-enhanced imaging.

**Figure 4:**
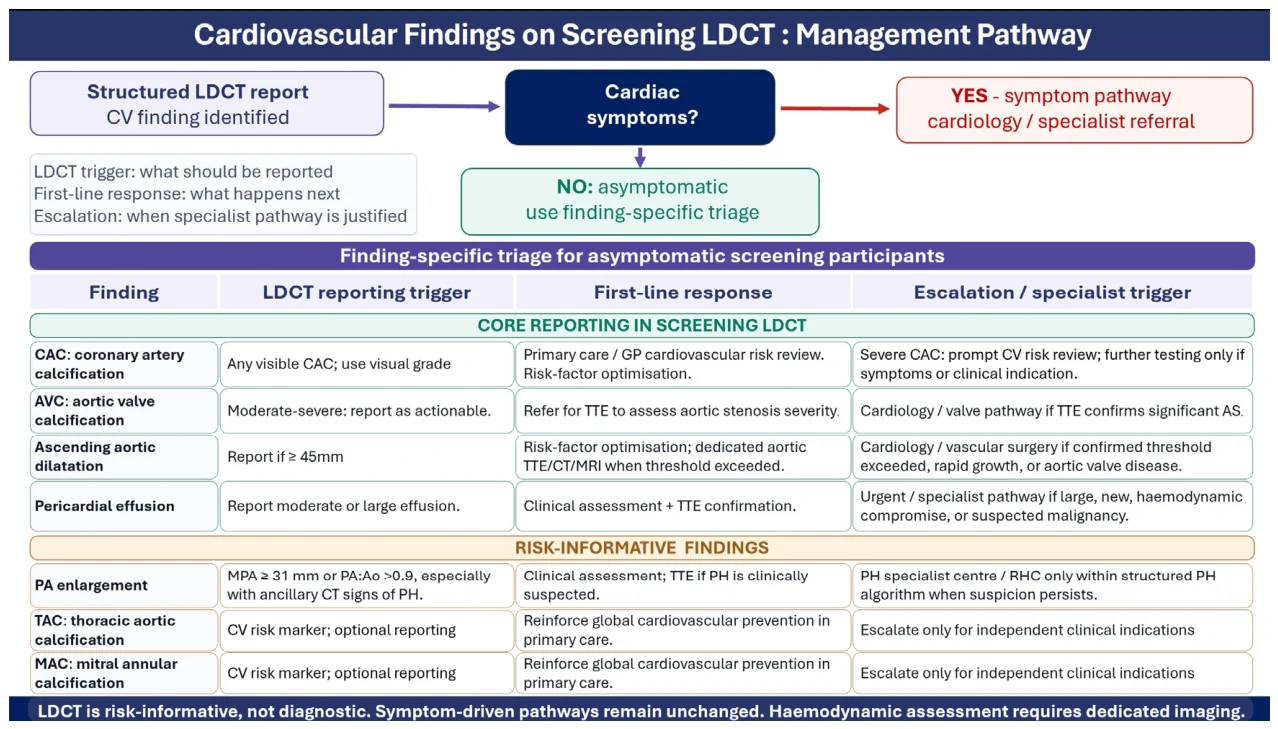
Management pathway for cardiovascular findings detected on screening LDCT. The flow chart outlines symptom-based referral and finding-specific triage for asymptomatic screening participants, distinguishing core reportable findings from risk-informative findings. Reporting triggers correspond to Table 1 and follow the ERS/ESTI statement (ascending aorta ≥ 45 mm; main pulmonary artery ≥ 31 mm or PA:Ao > 0.9). The pathway is the authors’ own synthesis of the ERS/ESTI statement [13], which adopts the ACR threshold for the pulmonary artery [12], and of the BSCI/BSCCT/BSTI consensus on reporting calcification [19]; the steps after referral follow the ESC guidelines on aortic disease [32] and pulmonary hypertension [35]. The pathway has not been validated prospectively. Abbreviations: AF, atrial fibrillation; AS, aortic stenosis; AVC, aortic valve calcification; CAC, coronary artery calcification; CAD, coronary artery disease; CT, computed tomography; CV, cardiovascular; ECG, electrocardiography; GP, general practitioner; LDCT, low-dose computed tomography; MAC, mitral annular calcification; MPA, main pulmonary artery; MRI, magnetic resonance imaging; PA, pulmonary artery; PA:Ao, pulmonary artery-to-aorta ratio; PH, pulmonary hypertension; RHC, right heart catheterisation; TAC, thoracic aortic calcification; TTE, transthoracic echocardiography.

## Data Availability

No new data were created or analysed in this study. Data sharing is not applicable to this article.

## Abbreviations

AI, artificial intelligence; AVC, aortic valve calcification; CAC, coronary artery calcification; CV, cardiovascular; EAT, epicardial adipose tissue; GP, general practitioner; LDCT, low-dose computed tomography; MAC, mitral annular calcification; PH, pulmonary hypertension.
